# 非小细胞肺癌中HIF-1α细胞亚群对淋巴结转移和术后复发的影响

**DOI:** 10.3779/j.issn.1009-3419.2026.106.05

**Published:** 2026-02-20

**Authors:** Fanghan CAO, Qianhui CHEN, Liying YANG, Miaoqing ZHAO, Xiaorong SUN, Ligang XING

**Affiliations:** ^1^250117 济南，山东第一医科大学（山东省医学科学院）研究生院; ^1^Department of Graduate, Shandong First Medical University (Shandong Academy of Medical Sciences) , Jinan 250117, China; ^2^250117 济南，山东第一医科大学（山东省医学科学院）附属肿瘤医院（山东省肿瘤防治研究院）放疗科; ^2^Department of Radiation Oncology, Shandong Cancer Hospital and Institute, Shandong First Medical University (Shandong Academy of Medical Sciences) (Shandong Cancer Research Institute), Jinan 250117, China; ^3^山东第一医科大学（山东省医学科学院）附属肿瘤医院（山东省肿瘤防治研究院）病理科; ^3^Department of Pathology, Shandong Cancer Hospital and Institute, Shandong First Medical University (Shandong Academy of Medical Sciences) (Shandong Cancer Research Institute), Jinan 250117, China; ^4^山东第一医科大学（山东省医学科学院）附属肿瘤医院（山东省肿瘤防治研究院）核医学科; ^4^Department of Nuclear Medicine, Shandong Cancer Hospital and Institute, Shandong First Medical University (Shandong Academy of Medical Sciences) (Shandong Cancer Research Institute), Jinan 250117, China

**Keywords:** 肺肿瘤, 无复发生存时间, 淋巴结转移, 预后, 乏氧诱导因子-1α, CD8^+^ T细胞, 肿瘤中心, Lung neoplasms, Recurrence-free survival time, Lymph node metastasis, Prognosis, Hypoxia-inducible factor-1alpha, CD8^+^ T cell, Tumor center

## Abstract

**背景与目的:**

非小细胞肺癌（non-small cell lung cancer, NSCLC）术后复发率较高，传统肿瘤原发灶-淋巴结-转移（tumor-node-metastasis, TNM）分期难以充分反映其生物学异质性，乏氧诱导因子-1α（hypoxia-inducible factor-1alpha, HIF-1α）在肿瘤进展及免疫微环境重塑中发挥重要作用，但其在不同淋巴结转移状态下的空间分布特征及对术后复发的预后意义尚不明确。本研究旨在探讨NSCLC患者原发灶中的表达HIF-1α的肿瘤细胞、CD4^+^ T细胞及CD8^+ ^T细胞的密度差异，以及对淋巴结转移及患者术后复发的影响。

**方法:**

回顾性收集2014年1月1日至2018年12月31日在山东第一医科大学附属肿瘤医院行根治性手术的256例NSCLC患者的术后石蜡包埋原发灶组织标本，构建包含肿瘤中心（tumor center, TC）和侵袭边缘（invasive margin, IM）区域的组织微阵列，并应用多重免疫荧光染色技术[HIF-1α/CD4/CD8/细胞角蛋白（cytokeratin, CK）/4',6-二脒基-2-苯基吲哚（4',6-diamidino-2-phenylindole, DAPI）]定量表达HIF-1α的肿瘤细胞（HIF-1α^+^CK^+^）、表达HIF-1α的CD4^+^ T细胞（HIF-1α^+^CD4^+^）及表达HIF-1α的CD8^+^ T细胞（HIF-1α^+^CD8^+^）的密度。采用*Mann-Whitney U*检验分析N_0_与N_1-2_组、N_1_与N_2_组中的细胞密度差异，*Cox*比例风险回归模型分析关键复发因子。

**结果:**

研究最终纳入256例IA-IIIB期NSCLC患者，中位随访时间为37.05个月，其中87例（34.0%）患者在随访期间复发。在NSCLC患者的TC及IM区域，与N_0_组相比，N_1-2_组HIF-1α^+^CK^+^细胞密度均更高（*P*分别为0.039、<0.001），HIF-1α^+^CD8^+^细胞密度均更低（*P*分别为<0.001、<0.001）；与N_1_组相比，N_2_组原发灶内TC区域和IM区域HIF-1α^+^CK^+^细胞、HIF-1α^+^CD4^+^细胞及HIF-1α^+^CD8^+^细胞的密度差异均无统计学意义（均*P*>0.05）。多因素*Cox*分析显示，在N_0_组患者中，TC区域低HIF-1α^+^CD8^+^密度是NSCLC患者复发的独立危险因素[风险比（hazard ratio, HR）=1.998，95%CI: 1.077-3.705，*P*=0.028]。在N_1_、N_2_组患者中，TC区域和IM区域HIF-1α^+^CK^+^密度、HIF-1α^+^CD4^+^、HIF-1α^+^CD8^+^密度与NSCLC患者复发无统计学关联。

**结论:**

在NSCLC患者中淋巴结转移与原发灶HIF-1α相关细胞亚群密度变化密切关联。原发灶TC区域中HIF-1α^+^CD8^+^细胞密度降低与N_0_期NSCLC患者术后复发显著关联，可作为术后风险分层的潜在免疫学指标。

## Abstract (en)

**Background and objective:**

Non-small cell lung cancer (NSCLC) is associated with a high rate of postoperative recurrence, and conventional tumor-node-metastasis (TNM) staging does not fully reflect its biological heterogeneity. Hypoxia-inducible factor-1alpha (HIF-1α) plays a critical role in tumor progression and remodeling of the tumor immune microenvironment. However, the spatial distribution of HIF-1α and its prognostic significance in the context of different lymph node metastatic states remain unclear. This study aimed to investigate the densities of HIF-1α-expressing tumor cells, CD4^+^ T cells, and CD8^+^ T cells in the primary tumors of NSCLC patients, and to assess their associations with lymph node metastasis and postoperative recurrence.

**Methods:**

Methods 256 formalin-fixed paraffin-embedded primary tumor specimens from NSCLC patients who underwent radical resection at Shandong First Medical University Affiliated Cancer Hospital between January 1, 2014 and December 31, 2018 were retrospectively collected. Tissue microarrays containing both tumor center (TC) and invasive margin (IM) regions were constructed and multiplex immunofluorescence staining [HIF-1α/CD4/CD8/cytokeratin (CK)/4',6-diamidino-2-phenylindole (DAPI)] were performed to quantitatively analyze the densities of HIF-1α-expressing tumor cells (HIF-1α^+^CK^+^), HIF-1α^+^CD4^+^ T cells and HIF-1α^+^CD8^+^ T cells. *Mann-Whitney U* tests were used to compare cellular density differences between N_0_ versus N_1-2_ groups and N_1 _versus N_2_ subgroups, while *Cox* proportional hazards regression models were employed to identify critical recurrence-associated factors.

**Results:**

The study ultimately included 256 patients with stage IA-IIIB NSCLC, with a median follow-up duration of 37.05 months, during which 87 cases (34.0%) experienced recurrence. Comparative analysis revealed that in both TC and IM regions, the N_1-2_ group exhibited significantly higher densities of HIF-1α^+^CK^+^ cells (*P* values: 0.039 and <0.001, respectively) and lower densities of HIF-1α^+^CD8^+^ cells (both *P* values: <0.001) compared to the N_0_ group, while no statistically significant differences were observed in the densities of HIF-1α^+^CK^+^ cells, HIF-1α^+^CD4^+^ cells, or HIF-1α^+^CD8^+^ cells between N_1 _and N_2_ subgroups within either TC or IM regions (all *P*>0.05). Multivariate *Cox *regression analysis showed that, among N_0_ patients, a low density of HIF-1α^+^CD8^+^ cells in the TC was an independent risk factor for recurrence in NSCLC patients [hazard ratio (HR)=1.998, 95%CI: 1.077-3.705, *P*=0.028]. In contrast, among N_1_ and N_2_ patients, the densities of HIF-1α^+^CK^+^ cells, HIF-1α^+^CD4^+^ T cells, and HIF-1α^+^CD8^+^ cells in both the TC and IM regions were not significantly associated with NSCLC recurrence.

**Conclusion:**

In patients with NSCLC, lymph node metastasis is closely associated with alterations in the densities of HIF-1α-related cellular subpopulations in the primary tumor. A reduced density of HIF-1α^+^CD8^+^ cells in the TC of the primary lesion is significantly associated with postoperative recurrence in N_0_-stage NSCLC patients and may serve as a potential immunological marker for postoperative risk stratification.

肺癌是全球发病率及病死率最高的恶性肿瘤之一，其中非小细胞肺癌（non-small cell lung cancer, NSCLC）占全部肺癌的85%-90%^[1]^。对于I、II期及部分IIIA期NSCLC患者，根治性手术切除是最佳治疗选择，但仍有30%-55%的患者在接受根治性手术切除后发生复发或转移导致治疗失败，提示仅依赖传统肿瘤原发灶-淋巴结-转移（tumor-node-metastasis, TNM）分期系统进行分期难以全面反映肿瘤的生物学行为^[2]^。因此，探索能够反映肿瘤免疫微环境特征并预测术后复发风险的生物标志物具有重要临床意义。

缺氧是大多数肿瘤的共同特征，在肿瘤进展及免疫逃逸过程中发挥关键作用。乏氧诱导因子-1α（hypoxia-inducible factor-1alpha, HIF-1α）是细胞应答低氧环境的核心转录调控因子^[3]^，可通过调控代谢重编程、血管生成及肿瘤侵袭转移等过程参与肿瘤进展^[4]^。已有研究^[5]^表明，HIF-1α不仅反映肿瘤缺氧状态，还在肿瘤免疫微环境重塑过程中发挥重要作用，包括调节免疫细胞募集、分化及功能状态，但其在肿瘤内不同细胞亚群中的空间分布及临床意义仍有待进一步阐明。

肿瘤浸润性淋巴细胞（tumor-infiltrating lymphocytes, TILs）是肿瘤免疫微环境的重要组成部分，其中CD8^+^ T细胞作为主要的抗肿瘤效应细胞，其浸润程度及在肿瘤组织中的空间分布特征与多种实体瘤的临床预后密切关联^[6,7]^。然而，肿瘤微环境中的缺氧状态可显著影响T细胞的功能表型及抗肿瘤效应，其免疫调控作用受肿瘤进展阶段和空间异质性的影响，使T细胞的预后意义呈现阶段性和区域性差异^[8]^。目前，关于HIF-1α表达的T细胞亚群在NSCLC中的临床意义仍缺乏系统性的研究，尤其是在不同淋巴结转移状态及肿瘤空间区域中的分布特征及其与患者预后的关系尚不明确。因此，将HIF-1α与传统T细胞标志物联合检测，可以区分处于不同功能状态的T细胞亚群，更精确地揭示肿瘤免疫微环境的动态变化。

基于此，本研究通过多重免疫荧光技术，定量分析NSCLC肿瘤中心（tumor center, TC）和侵袭边缘（invasive margin, IM）区域中HIF-1α^+^细胞角蛋白（cytokeratin, CK）^+^、HIF-1α^+^CD4^+^及HIF-1α^+^CD8^+^细胞密度和NSCLC患者根治术后复发的关系，并根据淋巴结转移状态进行分层分析，为术后风险分层及免疫治疗策略优化提供新的免疫学依据。

## 1 资料与方法

### 1.1 患者和样本

回顾性收集256例2014年1月1日至2018年12月31日于山东第一医科大学附属肿瘤医院行根治性手术的NSCLC患者的福尔马林固定石蜡包埋（formalin-fixed paraffin-embedded, FFPE）的原发灶组织学标本和临床信息。患者纳入标准：（1）美国癌症联合委员会第八版病理分期为IA-IIIB期的原发性NSCLC患者；（2）接受根治性肺叶切除术；（3）随访资料和病理标本的可获得性。排除标准：（1）合并其他恶性肿瘤；（2）术前接受过任何形式的抗肿瘤治疗；（3）缺乏完整的临床资料（相关临床资料从病历中获取）；（4）失访；（5）标本不完整。本研究经山东第一医科大学附属肿瘤医院伦理审查委员会批准（No.SDTHEC2022007013）。

### 1.2 组织微阵列（tissue microarry, TMA）制备

TMA由FFPE的患者组织构建，使用全自动TMA制作仪（TMA Grand Master, 3DHISTECH Ltd.）构建TMA（组织芯直径为1 mm）并进行连续切片（3 μm/层，LEICA RM2245），用于后续多重免疫荧光染色。对于每个TMA，使用包含同一患者的TC和IM的两个不同区域块。TC定义为肿瘤内部区域，IM定义为以正常组织与肿瘤组织交界处为中心，半径1 mm范围内的区域。所有切片均由至少两位研究者评估，其中包括一名具有副高级以上职称的病理科医师。

### 1.3 多重免疫荧光染色及图像分析

多重免疫荧光染色的指标包括CD4、CD8、HIF-1α^+^CK和4',6-二脒基-2-苯基吲哚（4',6-diamidino-2-phenylindole, DAPI）。将TMA切片置于65 ^o^C烤箱中2 h，并在二甲苯中脱蜡，分别在体积分数为100%、95%和70%梯度乙醇中进行再水化。95 ^o^C微波下切片在抗原修复液中加热15 min进行抗原提取。室温进行30 min抗原封闭。针对免疫标志物CD4、CD8、HIF-1α^+^CK，在切片上滴加一抗溶液[CD4 (Zsbio, 1:100), CD8 (Abcam, 1:500), HIF-1α (Abcam, 1:100), CK (Zsbio, 1:200)]200 μL室温孵育40-60 min。将切片用TSBT洗涤3次，5 min/次。一抗洗脱后，切片与二抗工作液室温孵育10 min。再次用TBST洗脱二抗，并滴加Opal染料[CD4 (1:100), CD8 (1:100), HIF-1α (1:100), CK (1:100)]200 μL，室温孵育10 min。最后滴加DAPI染料（1:100）200 μL，室温孵育8 min。

染色完成后，采用Akoya Polaris全景组织多光谱成像系统对TMA染色切片进行扫描以获得多光谱图像，其内置inForm软件进行光谱拆解去除组织自发荧光并分离各荧光信号。由经验丰富的病理科医师选取10-15张具有代表性的多光谱图像，对肿瘤巢和肿瘤间质进行人工标注以训练算法模型。随后，inForm软件基于组织形态特征，采用机器学习驱动的训练算法完成组织分割。细胞识别首先基于DAPI信号进行细胞核识别，并结合细胞形态参数完成单细胞分割；再根据CK、CD4、CD8以及HIF-1α的荧光信号强度及共定位关系进行细胞表型判读，从而导出各细胞亚群在TC和IM区域的细胞密度及空间分布数据^[9,10]^。随后使用R 3.6.3进一步处理inForm软件导出数据。

本研究中表达HIF-1α且同时表达CK的细胞定义为HIF-1α^+^CK^+^肿瘤细胞；表达HIF-1α且同时表达CD4或CD8的细胞分别定义为HIF-1α^+^CD4^+ ^T细胞和HIF-1α^+^CD8^+ ^T细胞。共表达的判定基于多重免疫荧光中同一细胞内HIF-1α与相应谱系标志物荧光信号的空间共定位。细胞密度以每1000个细胞内的特定细胞表型计数处理^[11]^。

### 1.4 统计学分析

采用SPSS 23.0及GraphPad Prism 10.1.2软件对数据进行统计学描述及分析。根据X-tile软件确定免疫相关参数的最佳截断值，并据此将患者分为高密度组（>截断值）和低密度组（≤截断值）。采用*Mann-Whitney U*检验分析N_0_与N_1-2_组间免疫指标差异，数据以中位数和四分位数形式呈现。采用单因素及多因素*Cox*比例风险回归模型分析复发有关的独立危险因素。在构建多因素模型前，对拟纳入变量进行了多重共线性检验。通过线性回归分析计算各变量的方差膨胀因子（variance inflation factor, VIF）及容差值评估共线性程度。多因素分析的显著变量进一步用于*Kaplan-Meier*生存曲线绘制及进行*Log-rank*检验。所有统计学检验均为双尾，*P*<0.05为差异有统计学意义。

## 2 结果

### 2.1 患者的临床病理特征

研究最终纳入256例IA-IIIB期NSCLC患者，年龄、性别、吸烟指数、组织学亚型、卡氏体能状态（Karnofsky performance status, KPS）评分、TNM分期、辅助放疗、辅助化疗及复发等临床资料见表1。患者以年龄≤65岁（66.0%）、男性（65.6%）、肺腺癌（64.5%）、KPS评分≥90分（71.5%）、I期疾病（53.5%）、辅助化疗（63.7%）及未行辅助放疗（85.5%）居多。中位随访时间为37.05个月（范围：26.80-47.50个月），共87例（34.0%）患者在随访期间复发，中位复发时间为28.02个月（范围：18.47-38.22个月）。在复发患者中，11例（12.6%）为单纯区域复发，56例（64.4%）为单纯远处转移，20例（23.0%）为区域复发合并远处转移。

**表1:** 256例NSCLC患者的基线特征

Characteristics	*n* (%)
Age (yr)	
≤65	169 (66.0)
>65	87 (34.0)
Gender	
Female	88 (34.4)
Male	168 (65.6)
Smoking index*	
<400	143 (55.9)
≥400	113 (44.1)
KPS score	
80	73 (28.5)
≥90	183 (71.5)
Histological type	
ADC	165 (64.5)
SCC	91 (35.5)
pT stage (AJCC v8)	
pT1	83 (32.4)
pT2	145 (56.6)
pT3	15 (5.9)
pT4	13 (5.1)
pN stage (AJCC v8)	
pN0	172 (67.2)
pN1	48 (18.8)
pN2	36 (14.0)
pN3	0 (0.0)
pTNM stage (AJCC v8)	
I	137 (53.5)
II	67 (26.2)
III	52 (20.3)
Adjuvant chemotherapy	
Yes	163 (63.7)
No	93 (36.3)
Adjuvant radiotherapy	
Yes	37 (14.5)
No	219 (85.5)
Recurrence	
Yes	87 (34.0)
No	169 (66.0)

*Smoking index means duration of smoking (years)×number of cigarettes smoked per year (cigarettes). NSCLC: non-small cell lung cancer; KPS: Karnofsky performance status; SCC: squamous cell carcinoma; ADC: adenocarcinoma; pTNM: pathological tumor-node-metastasis; AJCC: American Joint Committee on Cancer.

### 2.2 多色免疫荧光染色结果及细胞类型定义

图1A为1例肺鳞癌患者的代表性多色免疫荧光染色图像。染色结果显示，CK表达在细胞膜和细胞质中，CD4和CD8表达于细胞膜，HIF-1α表达于细胞核和细胞质。HIF-1α作为缺氧应答相关转录因子，在缺氧条件下可在肿瘤细胞、免疫细胞及部分非肿瘤源性上皮细胞中表达。因此，为提高HIF-1α表达判定的特异性，结合CK这一上皮来源标志物，定义的细胞类型包括：表达HIF-1α的肿瘤细胞（HIF-1α^+^CK^+^）、表达HIF-1α的CD4^+ ^T细胞（HIF-1α^+^CD4^+^）和表达HIF-1α的CD8^+ ^T细胞（HIF-1α^+^CD8^+^）（图1B）。

**图1:**
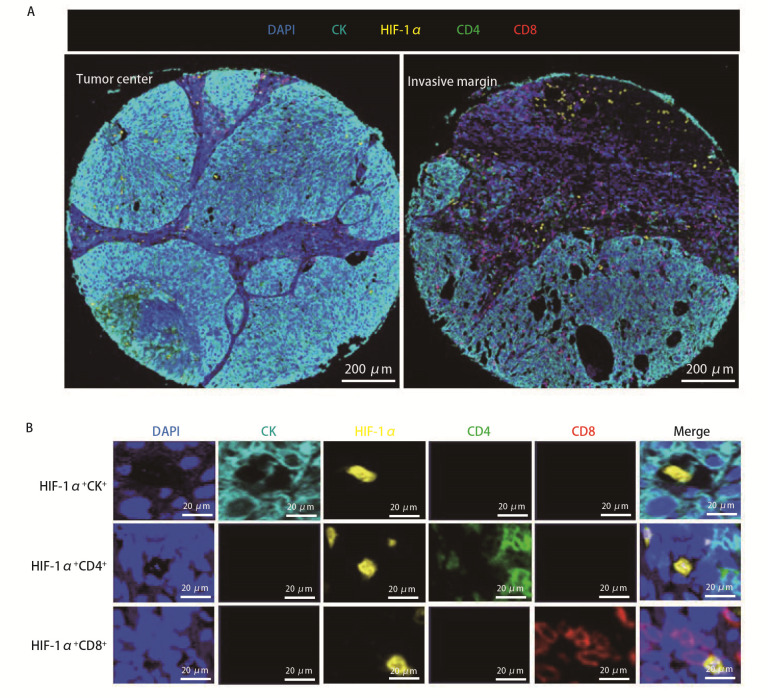
NSCLC组织多重免疫荧光染色结果及HIF-1α相关细胞类型的定义。A：NSCLC组织代表性多重免疫荧光图像；B：HIF-1α^+^CK^+^、HIF-1α^+^CD4^+^及HIF-1α^+^CD8^+^的染色示意图。DAPI：蓝色；CK：蓝绿色；HIF-1α：黄色；CD4：绿色；CD8：红色。

### 2.3 HIF-1α^+^CK^+^、HIF-1α^+^CD4^+^及HIF-1α^+^CD8^+^细胞密度与淋巴结转移的关系

根据患者术后病理淋巴结转移状态，将患者分为N_0_和N_1-2_组。*Mann-Whitney U*检验结果（表2）显示，无论是TC区域还是IM区域，与N_1-2_组相比，N_0_组中的HIF-1α^+^CK^+^细胞、HIF-1α^+^CD8^+^细胞的密度差异有统计学意义（均*P*<0.05），HIF-1α^+^CD4^+^细胞的密度差异无统计学意义（*P*>0.05）。进一步将淋巴结阳性患者分为N_1_和N_2_组。*Mann-Whitney U*检验结果显示，在TC和IM区域内，与N_2_组相比，N_1_组中的HIF-1α^+^CK^+^细胞、HIF-1α^+^CD4^+^细胞及HIF-1α^+^CD8^+^细胞的密度差异均无统计学意义（均*P*>0.05，表2）。

**表2:** N_1-2_与N_0_、N_2_与N_1_内的HIF-1α^+^CK^+^、HIF-1α^+^CD4^+^及HIF-1α^+^CD8^+^的密度差异（1/1000）

Item	N_0_	N_1-2_	*P*	N_1_	N_2_	*P*
TC HIF-1α^+^CK^+^	8 (4, 32)	19 (5, 50)	0.039	14 (3, 53)	22 (7, 45)	0.236
TC HIF-1α^+^CD4^+^	4 (1, 9)	4 (1, 7)	0.179	3 (1, 7)	4 (1, 7)	0.921
TC HIF-1α^+^CD8^+^	8 (3, 20)	2 (0, 6)	<0.001	1 (0, 5)	2 (0, 6)	0.691
IM HIF-1α^+^CK^+^	6 (2, 16)	15 (5, 32)	<0.001	13 (4, 26)	22 (5, 35)	0.216
IM HIF-1α^+^CD4^+^	2 (1, 9)	3 (1, 6)	0.863	3 (1, 7)	3 (1, 6)	0.581
IM HIF-1α^+^CD8^+^	10 (4, 27)	2 (1, 6)	<0.001	2 (1, 7)	2 (1, 6)	0.779

Data are expressed as the median with interquartile range. TC: tumor center; IM: invasive margin.

### 2.4 HIF-1α^+^CK^+^、HIF-1α^+^CD4^+^及HIF-1α^+^CD8^+^细胞密度与pTNM分期的关系

进一步分析了不同pTNM分期患者中HIF-1α相关细胞密度差异，结果（表3）显示，TC及IM区域内HIF-1α^+^CK^+^及HIF-1α^+^CD8^+^细胞密度在I与III期患者之间的差异具有统计学意义（均*P*<0.05），且部分指标在II与III期患者之间亦观察到显著差异。HIF-1α^+^CD4^+^细胞密度在不同pTNM分期间总体差异不显著，仅在IM区域中I与III期患者之间观察到统计学差异（*P*=0.036）。

**表3:** 不同pTNM分期患者中HIF-1α^+^CK^+^、HIF-1α^+^CD4^+^及HIF-1α^+^CD8^+^细胞密度的两两比较

Item	P_I vs II_	P_I vs III_	P_II vs III_
TC HIF-1α^+^CK^+^	0.405	<0.001	0.008
TC HIF-1α^+^CD4^+^	0.155	0.931	0.149
TC HIF-1α^+^CD8^+^	<0.001	<0.001	0.981
IM HIF-1α^+^CK^+^	0.001	<0.001	0.013
IM HIF-1α^+^CD4^+^	0.253	0.036	0.368
IM HIF-1α^+^CD8^+^	<0.001	<0.001	0.266

Pairwise comparisons were performed using Mann-Whitney U test, and all P values were two-sided.

### 2.5 HIF-1α^+^CK^+^、HIF-1α^+^CD4^+^及HIF-1α^+^CD8^+^对N_0_患者术后复发的影响

使用X-tile软件确定截断值，在IM区域，HIF-1α^+^CK^+^、HIF-1α^+^CD4^+^、HIF-1α^+^CD8^+^密度的截断值分别为23、0和2。在TC区域，HIF-1α^+^CK^+^、HIF-1α^+^CD4^+^、HIF-1α^+^CD8^+^密度的截断值分别为3、1和6。>截断值为高密度组，≤截断值为低密度组。进一步分析N_0_组患者瘤内IM区域和TC区域HIF-1α^+^CK^+^密度、HIF-1α^+^CD4^+^、HIF-1α^+^CD8^+^密度与NSCLC患者复发的关联性。单因素*Cox*分析（表4）结果显示，在N_0_患者中，年龄、性别、吸烟指数、组织学类型、pTNM分期、IM区域HIF-1α^+^CD4^+^密度、IM区域HIF-1α^+^CK^+^密度、IM区域HIF-1α^+^CD8^+^密度、TC区域HIF-1α^+^CK^+^密度、TC区域HIF-1α^+^CD4^+^密度与NSCLC患者复发无关联（均*P*>0.05）；TC区域低HIF-1α^+^CD8^+^密度与NSCLC患者复发有正向关联（*P*<0.05）。

**表4:** HIF-1α^+^CK^+^、HIF-1α^+^CD4^+^及HIF-1α^+^CD8^+^对N_0_患者术后复发的影响

Variables	Univariable analysis			Multivariable analysis	
	HR (95%CI)	*P*		HR (95%CI)	*P*
Age (≤65 yr vs >65 yr)	1.278 (0.673-2.428)	0.454			
Gender (Female vs Male)	1.399 (0.716-2.733)	0.326			
Smoking index* (<400 vs ≥400)	0.817 (0.446-1.497)	0.512			
Histological type (SCC vs ADC)	1.332 (0.714-2.482)	0.368		1.099 (0.530-2.279)	0.800
pTNM stage (AJCC v8)					
II vs I	1.504 (0.689-3.283)	0.305		0.508 (0.158-1.635)	0.256
III vs I	2.479 (0.868-7.081)	0.090		0.710 (0.210-2.402)	0.582
IM HIF-1α^+^CK^+ ^(Low vs High)	0.579 (0.291-1.153)	0.120			
IM HIF-1α^+^CD4^+ ^(Low vs High)	0.721 (0.099-5.243)	0.746			
IM HIF-1α^+^CD8^+ ^(Low vs High)	1.217 (0.513-2.890)	0.656			
TC HIF-1α^+^CK^+ ^(Low vs High)	0.724 (0.321-1.630)	0.435			
TC HIF-1α^+^CD4^+ ^(Low vs High)	1.698 (0.834-3.459)	0.145			
TC HIF-1α^+^CD8^+^(Low vs High)	2.091 (1.134-3.853)	0.018		1.998 (1.077-3.705)	0.028

*Smoking index means duration of smoking (years)×number of cigarettes smoked per year (cigarettes). HR: hazard ratio; CI: confidence interval.

在单因素分析基础上，将*P*<0.05的临床及免疫相关指标纳入多因素*Cox*回归模型，同时考虑临床相关性，将pTNM分期及组织学类型作为协变量进行校正分析。在构建多因素模型前，对拟纳入变量进行了多重共线性检验。结果显示，各变量VIF值均<2（范围：1.224-1.617），平均VIF值为1.359，容差值均>0.6，提示变量间不存在明显共线性风险，可同时纳入多因素*Cox*模型进行分析，结果（表4）显示，TC区域HIF-1α^+^CD8^+^密度降低是NSCLC患者复发的独立危险因素[风险比（hazard ratio, HR）=1.998，95%CI: 1.077-3.705，*P*=0.028]。*Kaplan-Meier*生存分析进一步表明，TC区域HIF-1α^+^CD8^+^低密度组患者的无复发生存时间（recurrence-free survival, RFS）显著降低（*P*=0.018），TC区域HIF-1α^+^CD8^+^低密度组的中位RFS为45.33个月（范围：0.77-65.27个月），HIF-1α^+^CD8^+^高密度组的中位RFS为47.36个月（范围：4.00-59.90个月）。

### 2.6 HIF-1α^+^CK^+^、HIF-1α^+^CD4^+^及HIF-1α^+^CD8^+^对N_1_、N_2_患者术后复发的影响

在N_1_、N_2_组患者中，单因素分析结果显示年龄、性别、吸烟指数、组织学类型、pTNM分期、TC区域和IM区域HIF-1α^+^CK^+^密度、HIF-1α^+^CD4^+^、HIF-1α^+^CD8^+^密度与NSCLC患者复发无统计学关联（均*P*>0.05，表5）。鉴于在单因素分析中未筛选出与复发显著关联的临床及免疫指标，且N_1_、N_2_亚组样本量相对有限，未进一步构建多因素*Cox*回归模型。

**表5:** HIF-1α^+^CK^+^、HIF-1α^+^CD4^+^及HIF-1α^+^CD8^+^对N1、N2组患者术后复发的影响

Variables	N_1 _univariable analysis			N_2 _univariable analysis	
	HR (95%CI)	*P*		HR (95%CI)	*P*
Age (≤65 yr vs >65 yr)	0.707 (0.277-1.802)	0.468		1.469 (0.500-4.316)	0.484
Gender (Female vs Male)	1.562 (0.592-4.124)	0.368		1.332 (0.559-3.173)	0.518
Smoking index* (<400 vs ≥400)	0.633 (0.257-1.561)	0.512		0.874 (0.401-1.904)	0.735
Histological type (SCC vs ADC)	1.326 (0.533-3.299)	0.544		1.098 (0.504-2.389)	0.814
pTNM stage (AJCC v8) (III vs II)	1.033 (0.299-3.563)	0.960		-	-
IM HIF-1α^+^CK^+ ^(Low vs High)	1.955 (0.642-5.954)	0.238		0.741 (0.341-1.612)	0.450
IM HIF-1α^+^CD4^+ ^(Low vs High)	1.095 (0.145-8.265)	0.930		2.379 (0.309-18.319)	0.405
IM HIF-1α^+^CD8^+ ^(Low vs High)	2.008 (0.796-5.061)	0.140		1.723 (0.793-3.742)	0.169
TC HIF-1α^+^CK^+ ^(Low vs High)	0.842 (0.277-2.558)	0.762		0.590 (0.078-4.441)	0.609
TC HIF-1α^+^CD4^+ ^(Low vs High)	1.187 (0.423-3.329)	0.745		0.809 (0.278-2.358)	0.698
TC HIF-1α^+^CD8^+ ^(Low vs High)	1.042 (0.343-3.163)	0.943		1.135 (0.476-2.706)	0.774

*Smoking index means duration of smoking (years)×number of cigarettes smoked per year (cigarettes).

## 3 讨论

许多早期NSCLC患者在根治性切除术后出现术后复发，而部分患者则存在相当长的RFS，其潜在分子机制仍有待进一步阐明。本研究主要探讨淋巴结阴性和阳性NSCLC患者原发灶中的表达HIF-1α的肿瘤细胞、CD4^+ ^T细胞及CD8^+ ^T细胞的密度差异，以及对淋巴结阴性及阳性患者术后复发的影响，表明其在预测NSCLC术后复发方面具有潜在的临床应用价值。

本研究发现，与N_0_组相比，N_1-2_组患者原发灶中HIF-1α^+^CK^+^肿瘤细胞密度增高，而HIF-1α^+^CD8^+^细胞密度降低。进一步的分层分析显示，仅在N_0_组患者中，TC区域HIF-1α^+^CD8^+^细胞密度降低与术后复发显著关联，并构成独立危险因素，而在N_1_、N_2_组患者中上述关联不再成立。这提示HIF-1α^+^CD8^+^细胞在NSCLC术后复发的预后价值具有明显的阶段性和空间特异性。

肿瘤缺氧是实体瘤发展的重要驱动因素之一，可通过激活HIF-1α信号通路促进代谢重编程、血管生成和转移侵袭^[12]^。既往研究表明，HIF-1α在NSCLC中的高表达与侵袭性增强和不良预后密切相关^[13]^，且在抗癌药物耐药机制激活中亦发挥重要作用^[14]^。在缺氧条件下，HIF-1α作为调节肿瘤低氧反应的关键转录因子，其稳定性和转录活性显著增强^[15]^，且其在肺癌组织中的表达水平明显高于良性组织^[16]^。因此，作为肿瘤细胞对缺氧环境的代谢适应，HIF-1α蛋白水平的持续升高被认为是癌症进展过程中的关键环节。本研究中，淋巴结阳性患者原发灶中HIF-1α^+^CK^+^肿瘤细胞密度显著升高，提示缺氧相关信号在转移发生前已在肿瘤局部被激活。该结果从临床角度支持HIF-1α作为NSCLC转移风险评估的潜在生物学标志，其在原发灶中的表达状态可能有助于早期识别具有高转移倾向的患者，并为个体化治疗策略的制定提供参考。

已有研究^[17]^表明，HIF-1α不仅反映肿瘤缺氧状态，还可在缺氧条件下调控CD8^+^ T细胞的糖酵解和干扰素-γ（interferon-gamma, IFN-γ）等效应分子的表达，从而有助于维持其抗肿瘤活性。CD8^+ ^T细胞是抗肿瘤免疫反应的核心效应细胞，其数量及功能状态直接影响肿瘤控制能力。在早期肿瘤微环境中，HIF-1α^+^CD8^+^细胞可能维持较高的功能性和抗肿瘤效应，因此其密度与良好预后有关。随着肿瘤进展及淋巴结转移的发生，肿瘤微环境经历持续缺氧和免疫编辑过程。已有研究^[8]^表明，HIF-1α可上调肿瘤细胞及肿瘤相关免疫细胞中程序性死亡配体1（programmed cell death ligand 1, PD-L1）的表达，从而通过程序性死亡受体1（programmed death protein 1, PD-1）/PD-L1通路抑制CD8^+^ T细胞效应功能并促进其耗竭。此外，HIF-1α介导的代谢重编程可增强肿瘤糖酵解活性，导致乳酸在肿瘤微环境中积累，乳酸和酸化环境进一步抑制CD8^+^ T细胞的增殖、细胞毒活性及细胞因子分泌，同时促进调节性T细胞（regulatory T cell, Treg）及髓系来源抑制性细胞（myeloid-derived suppressor cell, MDSC）等免疫抑制细胞的富集^[18]^。因此在N_1-2_阶段，尽管肿瘤组织中仍可检测到CD8^+^ T细胞及HIF-1α的表达，但这些细胞往往已处于功能性耗竭或免疫抑制状态，其数量难以真实反映抗肿瘤免疫效能，从而导致HIF-1α^+^CD8^+^细胞密度在N_1_及N_2_患者中与术后复发不再呈现显著关联。

本研究中，仅在TC区域而非IM区域观察到HIF-1α^+^CD8^+^细胞密度与复发的显著关联，提示肿瘤内免疫微环境存在显著的空间异质性。既往研究^[19]^提出“immune contexture”概念，强调免疫细胞在肿瘤不同空间区域（如TC与IM）的分布特征和功能状态对临床结局具有不同影响。免疫评分体系通过评估TC和IM区域中CD3^+^/CD8^+^ T细胞密度评估患者预后，表明肿瘤免疫空间差异本身具有独立的生物学和预后意义^[20]^。相比IM区域，TC区域更能反映肿瘤核心区域的缺氧压力和免疫抑制状态，这可能是TC区域HIF-1α^+^CD8^+^细胞在本研究中表现出更明确预后价值的重要原因。

此外，本研究中TC及IM区域的HIF-1α^+^CD4^+^细胞密度在不同分期中与临床结局均无显著统计学差异，这可能与CD4^+^ T细胞在肿瘤微环境中高度的功能异质性有关。不同于CD8^+^ T细胞直接介导细胞毒性杀伤，CD4^+^ T细胞由多种功能亚群组成，包括Th1、Th2、Th17及Treg，其在肿瘤微环境中既可促进抗肿瘤免疫反应，也可能参与免疫抑制过程^[21,22]^。因此，单纯以整体CD4^+^ T细胞密度作为评价指标，往往难以反映其真实的生物学效应，其预后意义受到亚群比例、功能状态以及空间分布等多重因素的共同影响。相比之下，HIF-1α^+^CD8^+^细胞作为直接的抗肿瘤效应细胞，其在TC区域的预后指示作用更为明确和一致。这一对比进一步突出了HIF-1α^+^CD8^+^细胞作为本研究核心发现的独特价值与潜在临床意义。

临床前研究^[23]^显示，功能干预研究如基因敲除或使用HIF-1α拮抗剂PX-478，证实在体内外均能降低肿瘤细胞的侵袭与转移能力，提示HIF-1α在转移发生中具有直接驱动作用。单细胞及空间图谱进一步在原发灶内鉴定出具有HIF-1α^high^表型的肿瘤细胞亚型，这些细胞富含侵袭、上皮-间质转化及趋化相关基因^[24]^，提示HIF-1α表达升高常为转移前的早期分子事件而非单纯的转移后被动适应。临床研究^[25]^显示，HIF-1α在NSCLC中与淋巴结转移和不良预后显著关联。因此，现有证据可支持HIF-1α作为NSCLC淋巴结转移的重要促发因子，可作为潜在的预后与治疗靶点。在此背景下，本研究提示N_0_期NSCLC患者中TC区域HIF-1α^+^CD8^+^细胞密度可作为术后复发风险分层的潜在指标，有助于识别传统分期下仍存在较高复发风险的患者群体，为术后辅助治疗决策提供参考。从临床转化角度看，HIF-1α^+^CD8^+^细胞密度降低可能标志着缺氧驱动、免疫抑制占优势的肿瘤微环境特征，这类患者单纯免疫治疗应答可能不佳，因此，HIF-1α^+^CD8^+^密度可用于筛选可能从免疫治疗或HIF-1α靶向联合治疗方案中获益的患者群体。

本研究存在局限性：（1）本研究为回顾性研究，可能存在选择偏倚；（2）样本量相对较小（尤其是N_1-2_患者），一定程度上限制了统计功效，仍需在更大规模且更具多样性的患者群体中验证研究结果；（3）多重免疫荧光主要用于检测蛋白表达水平，难以直接评估HIF-1α^+^CD8^+ ^T细胞功能状态；（4）HIF-1α的表达可能受缺氧程度、组织处理及固定时间等因素影响，单纯基于免疫荧光难以准确区分其动态活性状态。未来研究可结合功能实验及多组学手段进行深入验证。此外，可通过单细胞测序解析HIF-1α^+^CD8^+ ^T细胞的功能谱与克隆扩增情况，利用空间转录组学明确其与肿瘤及免疫抑制细胞的空间关系，并进一步讨论CD4^+ ^T及CD8^+ ^T细胞具体功能亚群对NSCLC患者淋巴结转移和复发的影响。这将有助于全面揭示HIF-1α在免疫调控与转移中的作用，并为NSCLC术后患者的精准分层治疗提供新的思路。

综上，本研究揭示了不同淋巴结转移状态对NSCLC患者原发灶中HIF-1α及T细胞密度的显著影响，提示HIF-1α相关细胞亚群及CD8^+ ^T细胞在NSCLC术后复发中的潜在作用，为术后复发风险评估及辅助治疗策略的制定提供了新的免疫学依据。
